# Pediatric Inﬂammatory Myoﬁbroblastic Tumor of Rectosigmoid Junction: A Case Report and Review of the Literature

**DOI:** 10.30699/ijp.2024.2003653.3122

**Published:** 2023-12-29

**Authors:** Mahsa Soti Khiabani, Maryam Monajemzadeh, Hojatollah Raji, Fatemeh Zamani, Mohammad Vaseie, Neda Pak

**Affiliations:** 1 *Children’s Medical Center, Pediatrics Center of Excellence, Tehran, Iran*; 2 *Department of Pediatric Emergency, Tehran University of Medical Sciences, Tehran, Iran*; 3 *Department of Pathology, Children Medical Center, Tehran University of Medical Sciences, Tehran, Iran.*; 4 *Pediatric Gastroenterology and Hepatology Research Center, Pediatrics Centre of Excellence, Children’s Medical Center, Tehran University of Medical Sciences, Tehran, Iran*; 5 *Department of Pediatric Surgery, Children’s Medical Center, Tehran University of Medical Sciences, Tehran, Iran*; 6 *Department of Radiology, Children Medical Center of Excellence, Tehran University of Medical Science, Tehran, Iran*; 7 *Advanced Diagnostic and Interventional Radiology Research Center, Tehran University of Medical Sciences, Tehran, Iran*; 8 *Emergency Medicine Department, Faculty of Medicine, Tehran University of Medical Sciences, Tehran, Iran*; 9 *Department of Radiology, Children Medical Centre of Excellence, Tehran University of Medical Sciences, Tehran, Iran*

**Keywords:** Computed tomography, Diagnosis, Inflammatory myofibroblastic tumor, X-ray

## Abstract

The occurrence of rectosigmoid junction inflammatory myofibroblastic tumor (IMT) is uncommon in children. This is a rare form of mesenchymal tumor, belonging to the category of soft tissue tumors, and can be found at any anatomical site from the central nervous system to the gastrointestinal tract.

Our patient was a 10-year-old male subject complaining of lack of defecation and constipation. The patient had decreased the frequency of defecation and constipation about two weeks before his referral and had not improved despite the use of laxatives. The abdomen was completely distended and there was no tenderness or guarding in the examination. Several airfluid levels are shown on the abdominal X-ray. In the ultrasound, free fluid was reported in the interlobular and pelvic spaces. The patient was transferred into the operating room. A tumor of the rectosigmoid junction was detected. Histopathologic studies showed evidence of IMT.

IMT is a rare neoplasm of unknown origin, which may occur in various sites of the body. Complete surgical removal is usually curative, but early detection of recurrence is required. Treatment options include chemotherapy, radiation therapy, and immunotherapy. Further investigations are needed to improve the understanding and management of this rare tumor.

## Introduction

Inflammatory myofibroblastic tumor (IMT) is an uncommon mesenchymal tumor of intermediate malignant potential that mostly involves pediatrics, adolescents, and young adults (1). It is also known as an inflammatory pseudotumor because it mimics malignancy, clinically and radiologically s (2). IMT can involve the lungs, abdomen, pelvis, and retroperitoneum, however, it may be seen in any part of the body. In most of the cases, IMT is a localized disease. Multifocal variant or metastatic disease is uncommon (3). Its etiology is unclear, but it is related to various factors such as trauma, infections, genetics, and autoimmune pathologies (4,5). In children, common sites of involvement included abdomen/pelvis (28%), head/neck region (22%), intrathoracic (22%), genitourinary (9%), bowel (6%) liver (6%), and musculoskeletal (6%) (4).

Here we report an uncommon case of IMT in a 10-year-old male who presented with constipation and gastrointestinal obstruction. The case affected the descending colonic region and a diagnosis of IMT was made histopathologically after the operation.

## Case Presentation

The patient was a 10-year-old male who came in with a complaint of lack of defecation and constipation. The patient presented with a decreased frequency of defecation and constipation of approximately t two weeks duration. The patient showed no improvement despite the use of laxatives. The patient's symptoms including abdominal pain and vomiting were reported to have gradually worsened. He reported an increase in the symptoms for two days before the visit. The patient had no stool or gas during the last 3 days.

In the initial examination of the patient, the vital signs were as follows:

Heart rate:120, respiratory rate:25, temp:37, blood pressure:100/70

In the examination, the abdomen was completely distended, and there was no tenderness or guarding.

**Fig. 1:**
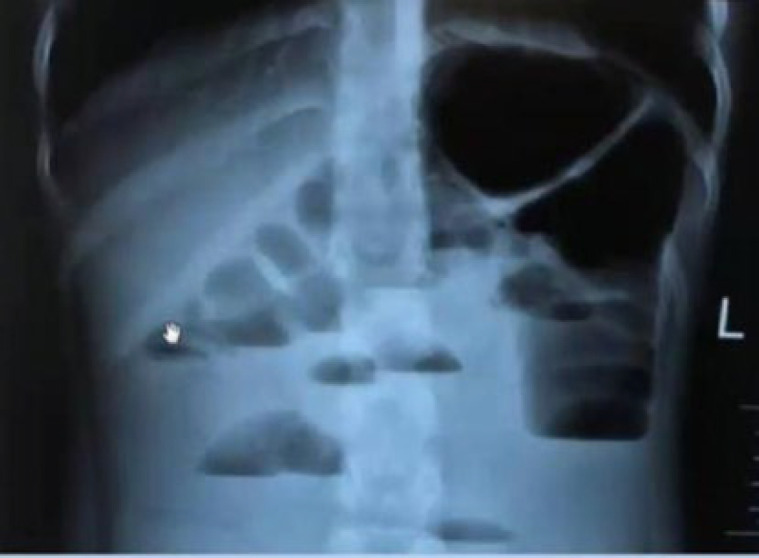
Abdominal X-ray showing multiple air-fluid levels

Before coming to the Children's Medical Center Hospital, the patient had been seen by another center and had undergone an abdominal X-ray and ultrasound, then he was referred to this center for further evaluations. Figure 1 shows the patient's graph. Free fluids have been reported in interlobular, subhepatic, right subphrenic, and pelvis areas during ultrasound examination. 

**Fig. 2:**
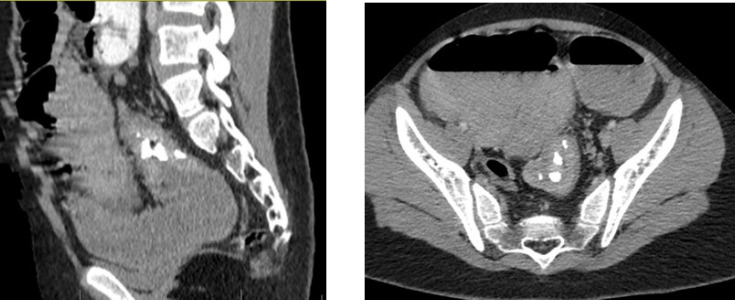
Abdomen and pelvic CT-scan with IV and oral contrast in axial plane (A) and sagittal plane (B) showing hyper-enhancing concentric wall thickening and a mass lesion with dysmorphic calcification

According to the above symptoms and results of the photographs, the patient was admitted with a primary diagnosis of colon obstruction. For this patient, initial treatments including appropriate fluid therapy, antibiotics prescriptions, and cardiorespiratory monitoring were carried out. Emergency surgical consultation and abdominal CT scan were requested for the patient. Figure 2 shows the CT scan features of the patient.

Evaluation of the CT scan features showed a complete obstruction of the colon due to the tumor. Then, the patient was transferred to the operating room as an emergency (10 hours after admission to the emergency room). Based on the pre-surgery laboratory data, evidence of elevated BUN with acidosis without electrolyte disturbances was noted. There was also a leukocytosis dominated by neutrophils.

During the surgery, the loops of the intestine and colon were found to be severely dilated. A tumor in the rectosigmoid junction was observed, which had caused complete expansion of the colon. The tumor showed attachment to the cecum, which was released and the tumor was resected. The left colectomy was performed. Terminal colostomy and Hartmann's colostomy were also performed. A pedunculated polyp was seen at the colectomy site, which was excised. 

After the surgery, urinary acid levels, alphafetoproteins, carcinoembryonic antigen, and cancer antigen 125 were found to be in a normal range. To determine the tumor’s nature, the samples were sent to the pathology department.

**Histopathologic Studies**

**Fig. 3:**
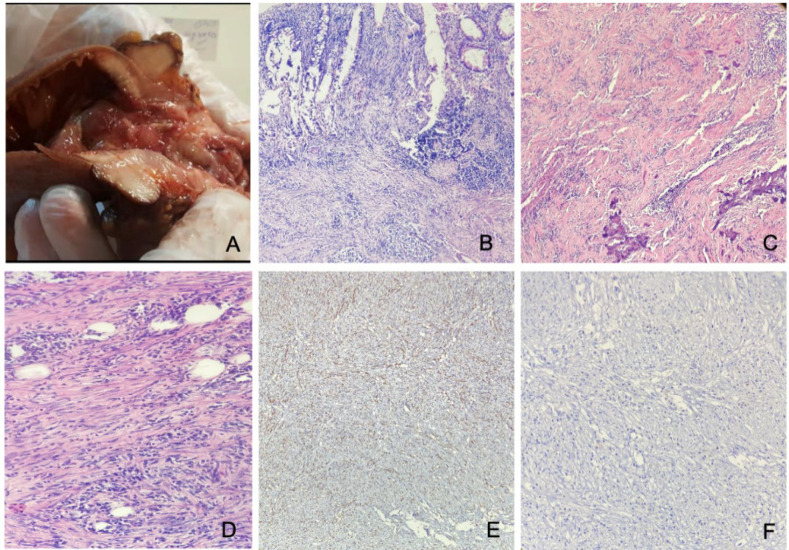
A: There was an intraluminal mass with a tan and homogenous cut surface. B-D: Microscopic examination showing a tumor within the mucosa and submucosa, composed of bland-looking spindle cells intermingled with lymphoplasma cells (A×100 and B, D ×400). E: The spindle cells are negative for CD34. F: The spindle cells are negative for anaplastic lymphoma kinase (ALK)

A formalin-fixed segment of the large intestine measuring 17.5 cm in length by 4.5 cm in greatest diameter was received. Proximal and distal surgical margins measured 4 cm and 2 cm in diameter, respectively. Through the opening, the intestinal lumen showed marked narrowing (almost obstructed). There was an indurated mass lesion with firm consistency and a homogeneous white cut surface measuring 2x1.8x1.4 cm. Focal osseous consistency was noted. Sections from the mass showed a neoplastic tissue mostly composed of spindle cells with fascicular arrangement, and minimal atypia in a variable cellular stroma containing abundant blood vessels. Infiltration of the lymphoplasma cells was prominently intermingled with the tumoral cells. These patterns were evident in nearly the whole thickness of the colonic wall involving mucosa, submucosa, and muscularis propria. Glandular architecture in involved areas shows degenerative changes. Feature of osseous metaplasia and calcification was seen focally. Mitotic figures were very rare and no atypical mitosis was observed. There was no obliterative phlebitis. Immunohistochemistry showed positive immunoreactivity with SMA and desmin in the tumoral cells. The stains of CD117, DOG1, S-100, CD34, and ALK showed negative results in the tumoral cells. Given the histopathologic and immunohistochemical features, a diagnosis of inflammatory fibroblastic tumor was made (Figure 3). About one month after the initial surgery, the patient was admitted for colostomy closure, and during abdominal pultrocolectomy and rectal mucosectomy, sleeve and pultrocoeloanal anastomosis was performed.

## Discussion

Inflammatory myofibroblastic tumor (IMT), was ﬁrst described in the lungs in 1937. It is also known as an inflammatory pseudotumor or plasma cell granuloma. IMT is a rare neoplastic lesion that is composed of a spindle cell population accompanied by an inflammatory infiltrate of plasma cells, lymphocytes, and eosinophils (6,7).

IMT can be located in different parts of the body such as the head and neck area, salivary glands, orbit, lung and liver, spleen, retrorectal space, soft tissues, peripheral nerves, heart, and genitourinary system (11). The urinary bladder is the most reported site of IMT (12,13). Pathogenesis of IMT remains unknown, although infection, trauma, and autoimmunity have been considered as potential origins. Infection has been associated with the development of IMT, including bacteria and viruses such as Epstein‑Barr virus, human herpes virus, *Bacillus sphaericus*, Mycobacterium tuberculosis, and hepatitis B (14-16).

Clinical presentation of IMT varies according to the site where the tumor originated. In the genitourinary system, IMT of the renal pelvis may exhibit symptoms such as hematuria, prolonged fever, and abdominal pain(12,17). Imaging features of IMTs of the renal pelvis are nonspecific and may vary in CT and MRI, presenting homo‑ or heterogeneity(12,18,19). Intra-abdominal IMT, same as our case, presents with an abdominal mass that is associated with an inﬂammatory response that manifests by fever and impaired growth and some symptoms related to the compression effect of the tumor such as abdominal pain and vomiting when it is located in the abdominal cavity. Rarely, the presentation may be a complicated profile such as intestinal obstruction (20-23). Patients with IMT have been also described to have laboratory evidence of an inflammatory response. These laboratory ﬁndings are hypochromic microcytic anemia, increased immunoglobulins, and elevated erythrocyte sedimentation rate, elevated thrombocyte counts (600.000 to 1.000.000). IMT may occur at any age, and both sexes are equally affected (24, 25).

The prognosis for IMT is generally good but depends on the tumor size and complete tumor removal. Overall survival rates are approximately 82 % in 3 years and about 74 % in 5 years. The local recurrence rate of pulmonary IMT following resection has been reported between 6.6% and 13%, occurring primarily in patients who had incomplete resection. Due to the multifocal nature of this disease entity rather than its metastatic spread, rare distant metastases have been also reported.

Follow-up radiological studies are strongly recommended, even if radiologic images look normal after surgery, and bronchoscopies should also be planned. For patients with this rare neoplastic lesion, more research needs to be done on the basic mechanisms of IMT and methods for efficient diagnosis and treatment. (24,26, 27).

Complete surgical removal is usually the most effective treatment for IMT, however, may be difficult to achieve in certain locations. Immunotherapies such as chemotaxis, radiation therapy, and immunotherapy are also used for IMT but their effectiveness is not yet known. In some cases, nonsteroidal anti-inflammatory drugs (NSAIDs), have been effective in reducing the size of the IMT, although the mechanism of action is unknown (6,24,28).

## Conclusion

IMT is a rare and uncommon neoplasm of unknown origin that can occur in various parts of the body. Complete surgical removal is usually curative, but close follow-up is necessary to recognize early recurrence. To better understand the management of this unusual tumor, further investigations are necessary to determine treatment options including chemotherapy, and radiation therapy. This should be considered based on a case-by-case.

Close radiological follow-ups are essential to detect any signs of recurrence or metastasis. To better understand pathogenesis as well as differentiation of aggression from as well nonaggression forms of IMT, additional studies are needed. In general, successful management of IMT requires early detection and complete surgical resection. 

## Conflict of Interest

The authors declare that they have no competing interests.
